# Efficacy and Safety of Non-Vitamin K Antagonist Oral Anticoagulants versus Vitamin K Antagonist Oral Anticoagulants in Patients Undergoing Radiofrequency Catheter Ablation of Atrial Fibrillation: A Meta-Analysis

**DOI:** 10.1371/journal.pone.0126512

**Published:** 2015-05-14

**Authors:** Giuseppe Santarpia, Salvatore De Rosa, Alberto Polimeni, Salvatore Giampà, Mariella Micieli, Antonio Curcio, Ciro Indolfi

**Affiliations:** 1 Division of Cardiology, Department of Medical and Surgical Sciences, “Magna Graecia” University, Catanzaro, Italy; 2 URT-CNR, Department of Medicine, Consiglio Nazionale delle Ricerche, Catanzaro, Italy; Universitätsklinikum des Saarlandes, GERMANY

## Abstract

**Background:**

Use of the non-vitamin K antagonist oral anticoagulants (NOACs) is endorsed by current guidelines for stroke prevention in patients with atrial fibrillation (AF). However efficacy and safety of NOACs in patients undergoing catheter ablation (RFCA) of AF has not been well established yet.

**Objectives:**

To perform a meta-analysis of all studies comparing NOACs and vitamin K antagonist oral anticoagulants (VKAs) in patients undergoing RFCA.

**Data Sources:**

Studies were searched for in PubMed and Google Scholar databases.

**Study Eligibility Criteria:**

Studies were considered eligible if: they evaluated the clinical impact of NOACs versus VKAs; they specifically analyzed the use of anticoagulants during periprocedural phase of RFCA; they reported clinical outcome data.

**Study Appraisal and Synthesis Methods:**

25 studies were selected, including 9881 cases. The summary measure used was the risk ratio (RR) with 95% confidence interval (CI). The random-effects or the fixed effect model were used to synthesize results from the selected studies.

**Results:**

There was no significant difference in thromboembolic complications (RR 1.39; p=0.13). Bleeding complications were significantly lower in the NOACs-treated arm as compared to VKAs (RR=0.67, p<0.001). Interestingly, a larger number of thromboembolic events was found in the VKAs-treated arm in those studies where VKAs had been interrupted during the periprocedural phase (RR=0.68; p=ns). In this same subgroup a significantly higher incidence of both minor (RR=0.54; p=0.002) and major bleeding (RR=0.41; p=0.01) events was recorded. Conversely, the incidence of thromboembolic events in the VKAs-treated arm was significantly lower in those studies with uninterrupted periprocedural anticoagulation treatment (RR=1.89; p=0.02).

**Limitations:**

As with every meta-analysis, no patients-level data were available.

**Conclusions and Implications:**

The use of NOACs in patients undergoing RFCA is safe, given the lower incidence of bleedings observed with NOACs. On the other side, periprocedural interruption of VKAs and bridging with heparin is associated with a higher bleeding rate with no significant benefit on onset of thromboembolic events.

## Introduction

Atrial fibrillation (AF) is the most common arrhythmia, its prevalence in the developed World is approximately 1.5–2% of the general population [1]. AF is associated with a fivefold increase in the risk of thromboembolic stroke [2], hence prophylactic anticoagulation is a cornerstone in the clinical management of AF. Thromboembolic prophylaxis by means of oral anticoagulants was shown to be very effective, leading to a 60% relative risk reduction of stroke, compared to placebo [3]. However, vitamin K antagonists, the only available drugs for long-term anticoagulation over the last 60 years, have some flaws: a) several days are needed to reach the full therapeutic effect; b) complex overlap with parenteral anticoagulants; c) narrow therapeutic window, which potentially exposes to the dreadful risk of bleeding; d) significant interaction with several drugs and food, making continuous monitoring of the therapeutic effect mandatory; e) common genetic variations of its metabolism. For these reasons, up to 65% of all patients at risk were not taking oral anticoagulants (OACs), while the international normalized ratio (INR) was out of range in a further 19% of patients [4]. Over the last years, non-vitamin K antagonist oral anticoagulant drugs (NOACs) have been developed, including direct thrombin inhibitors (dabigatran) and factor Xa inhibitors (rivaroxaban, apixaban). Their therapeutic use for prevention of cardio-embolic complications was validated in recent large phase III trials, demonstrating their non-inferiority, and even superiority, in some cases, to warfarin [5,6,7]. Therefore, use of NOACs is currently recommended by guidelines, along with vitamin K antagonists, for stroke prevention in patients with non-valvular atrial fibrillation [8]. However, giving the relatively short experience with this new class of OACs in clinical practice, clinicians still face uncertainties on their efficacy and safety in patients undergoing catheter ablation of atrial fibrillation, due to the lack of clinical evidence in this specific population. We therefore aim to systematically evaluate all available evidence on the use of NOACs versus vitamin K antagonist oral anticoagulants (VKAs)in patients with atrial fibrillation allocated to a rhythm management therapy by radiofrequency catheter ablation (RFCA) using a meta-analytic approach to synthesize the results from all available studies. In particular, specific objectives of the present meta-analysis were: 1) evaluate the efficacy and safety of NOACs versus VKAs in patients with atrial fibrillation undergoing RFCA, and 2) test whether peri-procedural interruption of VKAs with heparin bridging could affect the clinical outcome of patients undergoing RFCA.

## Methods

### Search strategy and study selection

Published studies comparing NOACs to VKAs were searched for within PubMed and Google Scholar electronic databases up to February 28^th^ 2015. The following key words were used: “dabigatran” “rivaroxaban” “apixaban” “atrial fibrillation”, and “radiofrequency catheter ablation”. Time of publication was not a limiting criterium for our analysis, while an English language restriction was made. All reports were independently screened by two investigators for eligibility. In addition, references from the selected articles were scanned for eligible studies, as already described before [9]. Any disagreement was resolved through discussion. All included studies were thoroughly checked and classified to exclude duplicity of data. Studies were considered eligible if: a) they evaluated the clinical impact of NOACs versus VKAs; b) they specifically analyzed the use of anticoagulants during periprocedural phase of RFCA; c) they reported clinical outcome data (stroke, transient ischemic attack or systemic embolism, major or minor bleedings). Exclusion criteria were (just one was sufficient for study exclusion): duplicate publication, pre-specified endpoint measure not specified.

### Data abstraction and validity assessment

Baseline characteristics and numbers of events were extracted from all studies by two independent reviewers. Divergences were resolved by consensus after discussion. The following data were abstracted: year of publication, study location, number of patients, study design, clinical outcome data (stroke, systemic or pulmonary embolism, transient ischemic attack, minor bleedings and major bleedings), and baseline patients’ characteristics. In studies in which propensity score matching was applied to generate a cohort of two groups with balanced baseline data, only “after propensity score matching” data were considered in meta-analysis. Outcome data were separately analyzed for those studies reporting independent comparisons between multiple groups. Selection and data abstraction were performed according to the PRISMA statement (S1 Table) [10].

### Statistical analysis

The summary measure used was the risk ratio (RR) with 95% confidence interval (CI). The random-effects model described by DerSimonian and Laird was used to combine the results from single studies [11]. This model calculates a weighted average by incorporating within-study and between-study variations. The Mantel-Haenszel method (fixed effect model) was also used to assess the effect of model assumptions on our conclusions, depending on study heterogeneity [12]. Forest plots were used to graphically display the results of the meta-analysis, as already previously described [13]. Briefly, the left column lists the names of the studies. The right column is a plot of the measure of effect (risk ratio) for every study (represented by a square), incorporating 95% confidence intervals, represented by horizontal lines. The area of each square is proportional to the study's weight in the meta-analysis. Finally, the central column shows the calculation results of the meta-analysis. The overall effect measure is reported on the bottom line of the plot (diamond), where the width indicates the confidence interval. Heterogeneity was assessed with the Cochrane Q test using a chi-squared function (p values < 0.10 were considered significant). In addition, I^2^ values were calculated to estimate the proportion of variation in the effect size attributable to between-study heterogeneity. An I^2^ value > 20% was considered significant. A random-effects model was preferred if appropriate, with a two-sided p value < 0.05 considered significant, as previously described [14]. Small study effects were evaluated through graphical inspection of funnel plots and statistically using the Egger’s test [15]. Analysis was performed by means of Excel spreadsheets and RevMan 5.

## Results

### Study characteristics

**Fig 1:**
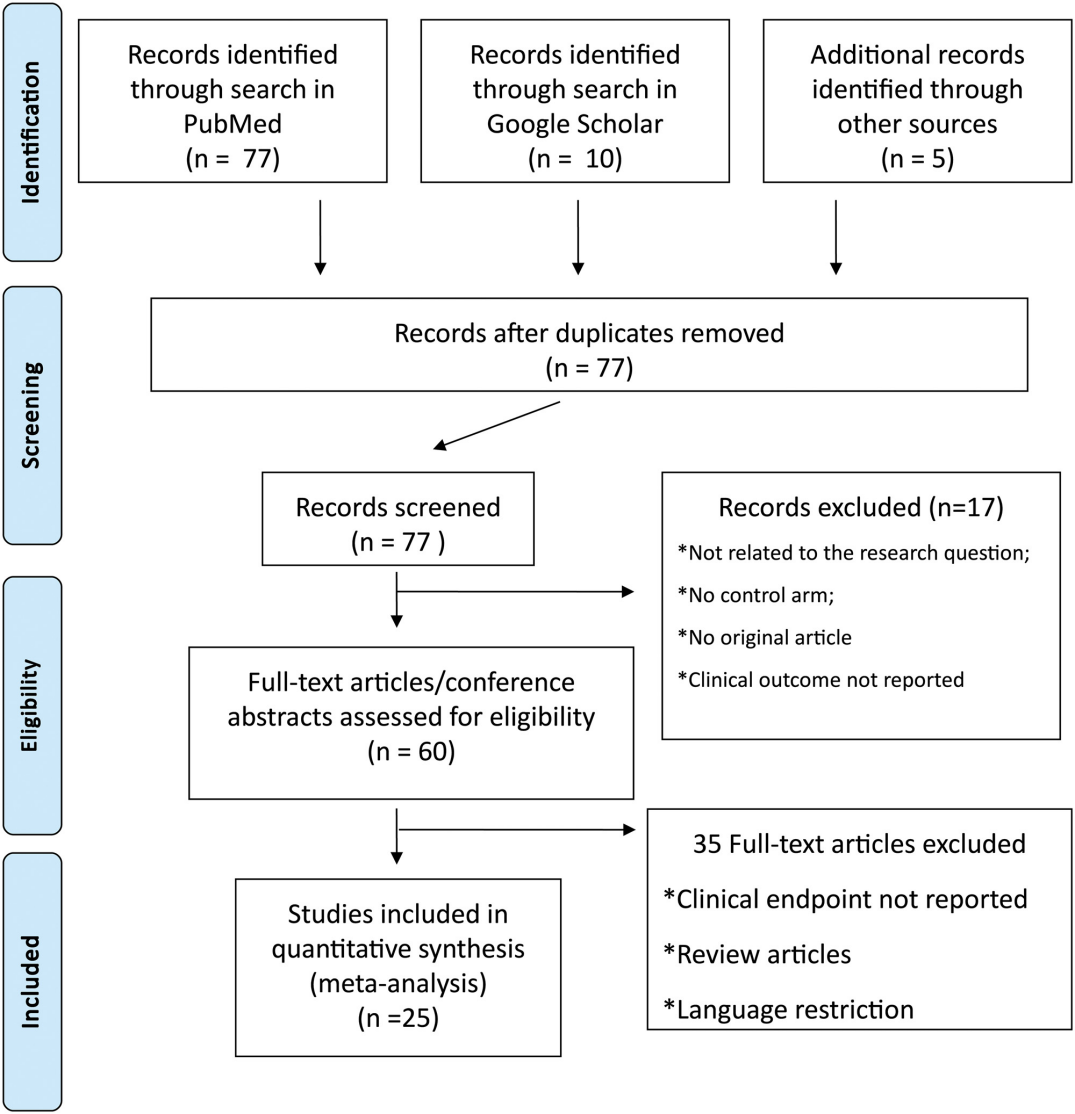
Study selection flow chart.

Our database search retrieved a total of 77 entries from PubMed, 10 from Google Scholar and 5 from congress abstracts. Overall, a total of 77 studies were available after removal of duplicates, which were screened with exclusion of further 52 studies based on pre-specified criteria. In particular, most studies were excluded at this stage while they either did not report the clinical endpoints, they were review articles, they were written in non-English language. Finally, a total of 25 studies fulfilled the inclusion criteria and were selected for this meta-analysis. The stepwise selection process is illustrated in Fig 1. Details of the selected studies are showed in table 1. Thromboembolic and bleeding risk profiles were similar in both study arms for all studies, except three [16,17,18]. Data on the CHADS_2_ or the CHA_2_DS_2_-VASc score and the HAS-BLED score are showed in S2 Table. Endpoints’ definition for all selected studies are reported in S3 Table.

**Table 1:** Details of the selected studies included in the Meta-analysis.

STUDY	YEAR	LOCATION	N	SOURCE TYPE	STUDY DESIGN	PRIMARY END POINT	OTHER END POINTS	FUP
***Kaseno [20]***	2012	Japan	211	Journal Article	Retrospective non-blinded single centre study	Efficacy and safety of peri-procedural dabigatran in AF ablation.	Death, Stroke, Major and Minor bleeding	30 days
***Snipelisky [21]***	2012	USA	156	Journal Article	Retrspective single centre study	Provide information on the use of dabigatran in patients undergoing atrial arrhythmias ablation	Major and minor Bleeding	30 days
***Lakkireddy [37]***	2012	USA	290	Journal Article	Prospective study	Feasibility and safety of continuing dabigatran during AF ablation.	Major and minor bleeding, thromboembolism	30 days
***Konduru [22]***	2012	USA	76	Journal Article	Retrospective study	Compare heparin administration and degree of anticoagulation achieved in patients treated with dabigatran and warfarin before AF ablation	Stroke, Major Bleeding	30 days
***Mendoza [28]***	2012	USA	119	Conference Abstract	Retrospective study	Feasibility and safety of dabigatran in the setting of AF ablation.	Stroke, major and minor bleeding	30 days
***Rowley [29]***	2012	USA	282	Conference Abstract	Retrospecrive study	Feasibility and safety of dabigatran for AF ablation.	Stroke, major and minor bleeding	30 days
***Ellis [31]***	2012	USA	171	Conference Abstract	Retrospective study	Feasibility and safety of dabigatran for AF ablation.	Stroke, major and minor bleeding	30 days
***Pavaci [30]***	2012	Germany	54	Conference Abstract	Retrospective study	Feasibility and safety of dabigatran for AF ablation.	Stroke, major and minor bleeding	30 days
***Kaiser [23]***	2013	USA	257	Journal Article	Retrospective study	Feasibility and safety of dabigatran for AF ablation.	Stroke and Major bleeding	90 days
***Haines [24]***	2013	USA, Canada	404	Journal Article	Retrospective multicentre pilot trial	Feasibility and safety of dabigatran for AF ablation.	Freedom from stroke and major bleeding	-
***Imamura [38]***	2013	Japan	227	Journal Article	Prospective observational single centre study	Feasibility and safety of dabigatran for AF ablation.	Stroke, major and minor bleeding	30 days
***Maddox [16]***	2013	USA	463	Journal Article	Retrospective single centre study	Feasibility and safety of dabigatran for AF ablation.	Stroke, major and minor bleeding	90 days
***Nin [36]***	2013	Japan	90	Journal Article	Randomized single centre study	Feasibility and safety of dabigatran for AF ablation.	Stroke, major and minor bleeding	12 months
***Bernard [25]***	2013	USA	274	Conference Abstract	Retrospective single centre study	Compare the feasibility and safety of dabigatran, rivaroxaban and warfarin, used for peri-procedural anticoagulation in AF ablation	Stroke, major and minor bleeding	30 days
***Kim [26]***	2013	USA	763	Journal Article	Retrospective, not randomized case control study	Compare the feasibility and safety of dabigatran and warfarin used for peri-procedural anticoagulation in AF ablation	Thromboembolism, major and minor bleeding	90 days
***Bassiouny [39]***	2013	USA	688	Journal Article	Prospective, not randomized trial	Evaluate the use of dabigatran for perioperative anticoagulationinpatients undergoing AF ablation compared with uninterrupted warfarin therapy	Thromboembolism, major and minor bleeding	90 days
***Yamaji [27]***	2013	Japan	503	Journal Article	Retrospective single-centre study—partially randomized study	Evaluate the feasibility and safety of dabigatran in the setting of AF ablation	Stroke, major and minor bleeding	90 days
***Ichiki [18]***	2013	Japan	210	Journal Article	Prospective, not randomized study	Compare the feasibility and safety of dabigatran and warfarin used for peri-procedural anticoagulation in AF ablation by investigation of asymptomatic cerebral microthromboembolism detected by MRI	Major bleeding	24 hours
***Piccini [19]***	*2013*	America Australia Europe	321	Journal Article	Post-hoc multi-centre analysis	Composite of all strokes (both ischemic and hemorrhagic) and systemic embolism. Safety endpoint was major or non-major clinically relevant bleeding.	cardiovascular death, all-cause death, composite of stroke, systemic embolism, or CV death, composite of stroke, systemic embolism, or all-cause death.	2.1 years
***Stepanyan [32]***	2014	USA	301	Journal Article	Retrospective single centre study	Evaluate the feasibility and safety of dabigatran and rivaroxaban in the setting of AF ablation	Stroke, TIA, major and minor bleeding	30 days
***Providencia [40]***	2014	France	556	Journal Article	Prospective not randomized single-centre study	Assess the efficacy and safety of dabigatran and rivaroxaban in patients referred for AF catheter ablation compared with VKA.	All cause of death,Thromboembolism, major and minor bleeding	30 days
***Arshad [33]***	2014	USA	882	Journal Article	Retrospective multicentre study	Compare safety and efficacy of different periprocedural anticoagulation strategies in catheter ablation of atrial fibrillation	Stroke and minor bleeding	30 days
***Winkle [17]***	2014	USA	1726	Journal Article	Retrospective single centre study	Evaluate peri-procedural AF ablation complications using different OACs	Thromboembolism and bleeding	30 days
***Dillier [34]***	2014	Germany	544	Journal Article	Retrospective single centre study	Evaluate peri-procedural left atrial ablation complications using different OACs	Thromboembolism, bleeding and death	Hospitalization Time
***Kaess [35]***	2015	Germany	315	Journal Article	Retrospective single centre study	Evaluate peri-procedural left atrial ablation complications using different OACs	Thromboembolism, bleeding and death	Hospitalization Time

Periprocedural anticoagulation was managed in different ways in the various studies. In fact, VKAs administration was interrupted in some studies and kept uninterrupted in other studies. In the first case, the anticoagulation treatment was stopped 2 to 5 days before the RFCA procedure and replaced by low molecular weight heparin (LMWH) bridging. In the remaining studies VKAs administration remained uninterrupted throughout the procedural phase, keeping the INR value within the 2–3 target. On the contrary, periprocedural NOAC management was homogeneous across almost all studies included. The NOAC assumption was stopped 12 to 30 h before the RFCA procedure without any LMWH bridging and started again 6 to 24 h after. Finally, in both study arms (VKAs and NOACs) intraprocedural anticoagulation was achieved with the administration of a weight-based unfractionated heparin bolus just after crossing the interatrial septum to the left atrium with the transseptal sheath. After bolus administration, iv heparin infusion (1000 IU/h) was continued during the procedure, keeping the activating clotting time (ACT) between 250 and 400 seconds. Of all selected studies, one is a post-hoc analysis of a large randomized trial [19]. It evaluated efficacy and safety of rivaroxaban in AF patients treated with either cardioversion or RFCA. Sixteen studies [16,17,20,21,22,23,24,25,26,27,28,29,30,31,32,33] are retrospective analyses including patients that had undergone RFCA of AF with periprocedural anticoagulation utilizing warfarin and dabigatran or rivaroxaban. Two studies [34,35] are retrospective analyses including patients that had undergone left atrial RFCA with periprocedural anticoagulation utilizing phenprocoumon and rivaroxaban or apixaban. One study is a small-randomized prospective trial that evaluated the safety and efficacy of dabigatran in comparison with warfarin in the setting of RFCA of AF [36]. The remaining five studies are prospective, non-randomized, observational studies analyzing the safety and efficacy of dabigatran/rivaroxaban in comparison with warfarin in patients undergoing RFCA of AF [18,37,38,39,40]. Study quality assessment performed according to the PRISMA statement.

### Clinical events

The 25 studies selected for this meta-analysis included a total of 9881 patients (4229 treated with NOACs and 5652 treated with VKAs). A total of 79 embolic events (35 in the NOACs arm and 44 in the VKAs arm) and a total of 533 bleeding events (182 in the NOACs arm and 351 in the VKAs arm) were registered.

### Stroke and thromboembolism

**Fig 2:**
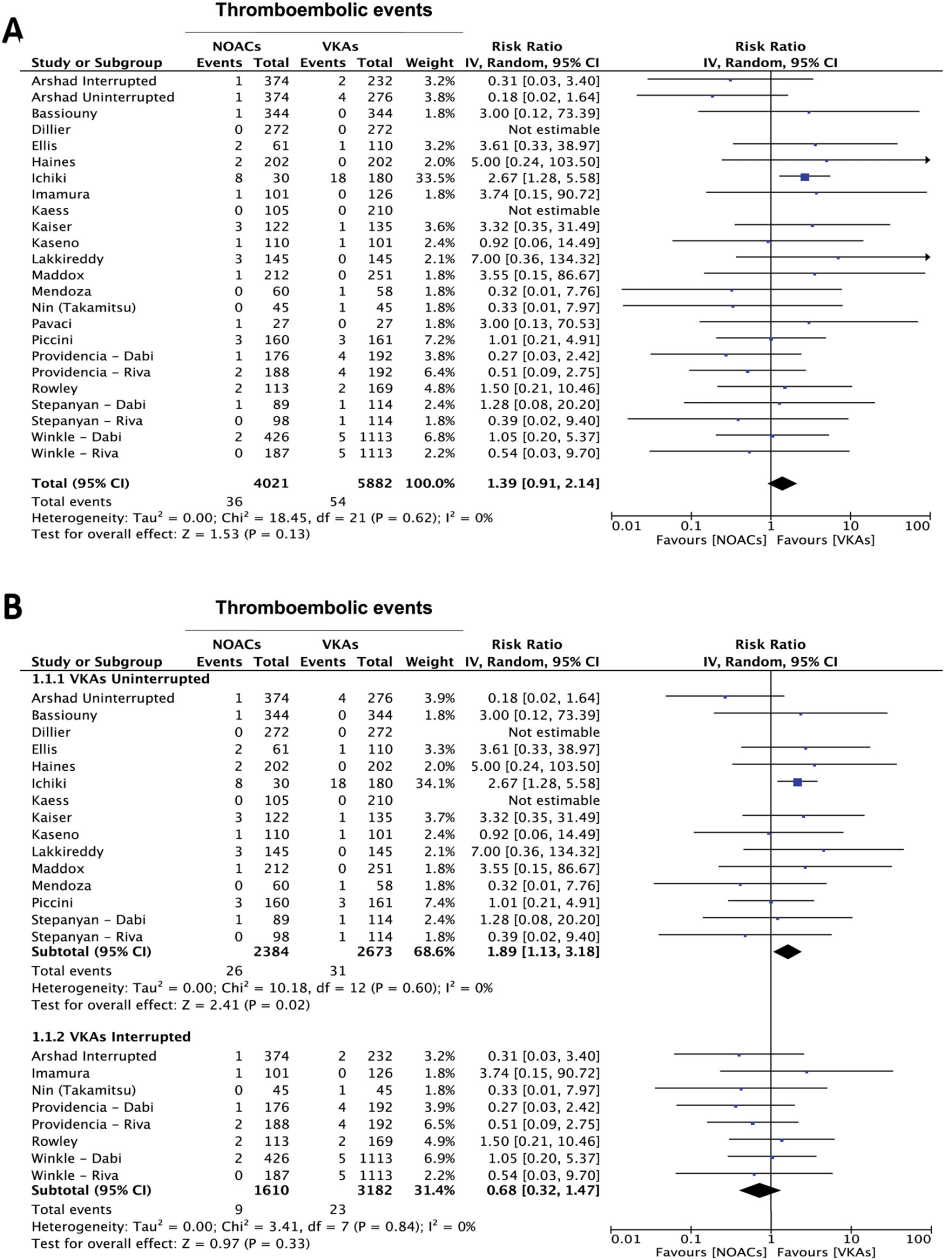
Meta-analysis of difference in thromboembolic events. Panel A: forest plot and summary effect of the difference in the incidence of thromboembolic events. Panel B: difference in the thromboembolic events is shown in the subgroups of patients undergoing interrupted vs. uninterrupted periprocedural VKAs.

**Fig 3:**
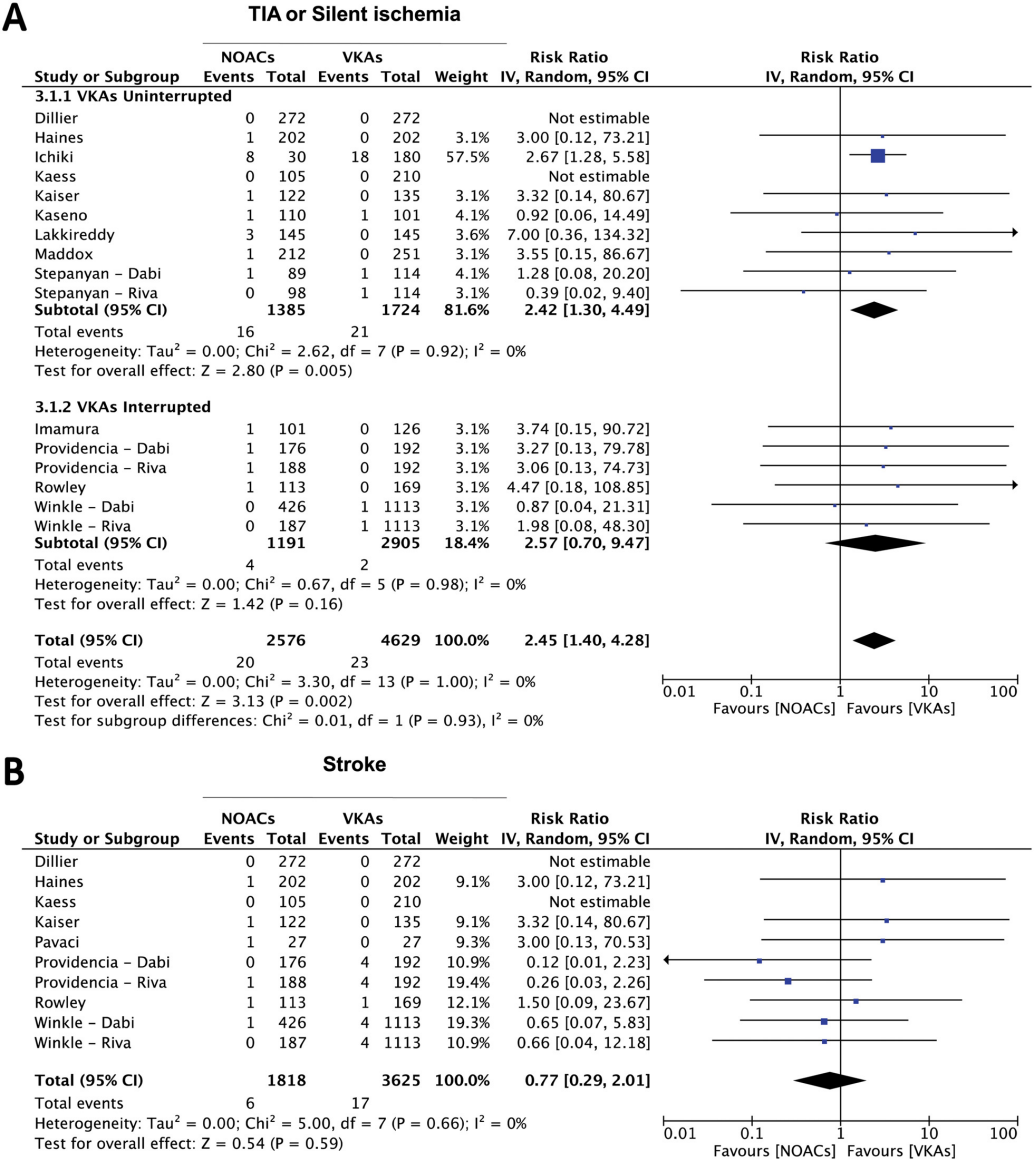
Meta-analysis of difference in TIA and Stroke. Panel A: forest plot and summary effect of the difference in the incidence of TIA or silent ischemia. Panel B: forest plot and summary effect of the difference in the incidence of Stroke.

Analyzing the results from all studies reporting on thromboembolic events, no significant difference in the incidence of this endpoint was found (RR 1.39, 95%CI 0.91–2.14; p = 0.13) (Fig 2A). Of note, the sensitivity analysis revealed that this result was constant across all selected studies and the overall effect didn’t significantly change even after removal of single studies, with one exception. In fact, we evaluated the influence of removal of each of the two studies where the two treatment arms were slightly unbalanced in the baseline predicted thromboembolic risk. Indeed, removal of the study by Kaiser and colleagues [23] had no influence on the cumulative result while removal of the study by Providencia and colleagues [40] significantly affected the cumulative result, with a significantly lower incidence of the endpoint in the VKAs-treated group (RR 1.61, 95%CI 1.02–2.52; p = 0.04). To further investigate the origin of the observed incidence of the endpoint, we performed another subgroup analysis, dividing the studies in two groups: the first (interrupted vitamin K antagonist oral anticoagulants, IVKAs) including all studies in which periprocedural VKAs interruption with heparin bridging was performed; the second (uninterrupted vitamin K antagonist oral anticoagulants, CVKAs) including the studies in which no periprocedural interruption of VKAs treatment was applied. Of note, this subgroup analysis showed a significant difference in the incidence of thromboembolic events in favor of VKAs, that was limited to the subgroup of studies in which the VKAs treatment had not been interrupted during the periprocedural phase (RR = 1.89 95% CI 1.13–3.18; p = 0.02), while no significant difference was found in the interrupted group (RR = 0.68 95% CI 0.32–1.47; p = 0.33) (Fig 2B). Furthermore, since 22 studies had used dabigatran and 6 rivaroxaban, we performed a further subgroup analysis, which did not show a substantial difference between the study subgroups using different NOACs (S1 Fig). Accordingly, comparison between the study subgroups didn’t show a statistically significant difference (p = 0.30). In addition to the composite endpoint, enough data were available from the selected studies, to look after the overall affect on single clinical endpoints. Interestingly, a similar result was obtained analyzing the summary effect on the cumulative endpoint of transient ischemic attack (TIA) or silent cerebral ischemia (SCI), where a significant difference was found in favor of the VKAs-treated group (RR = 2.45 95% CI 1.40–4.28; p = 0.002) (Fig 3A). This result was driven by the subgroup of studies in which VKAs were not interrupted during the periprocedural phase (RR = 2.42 95% CI 1.30–4.49; p = 0.005), while the difference in favor of VKAs lost its statistical significance when the analysis was restricted to the studies in which the oral anticoagulation with VKAs had been interrupted and bridged with heparin during the periprocedural phase (RR = 2.57 95% CI 0.70–9.47; p = 0.16) (Fig 3A). A smaller number of studies provided data on the isolated endpoint of ischemic stroke [23,24,28,29,30,40]. Summing the evidence from these studies, no difference was found between NOACs and VKAs-treated arms (RR 0.77, 95% CI 0.29–2.01, p = 0.59) (Fig 3B).

### Bleedings

**Fig 4:**
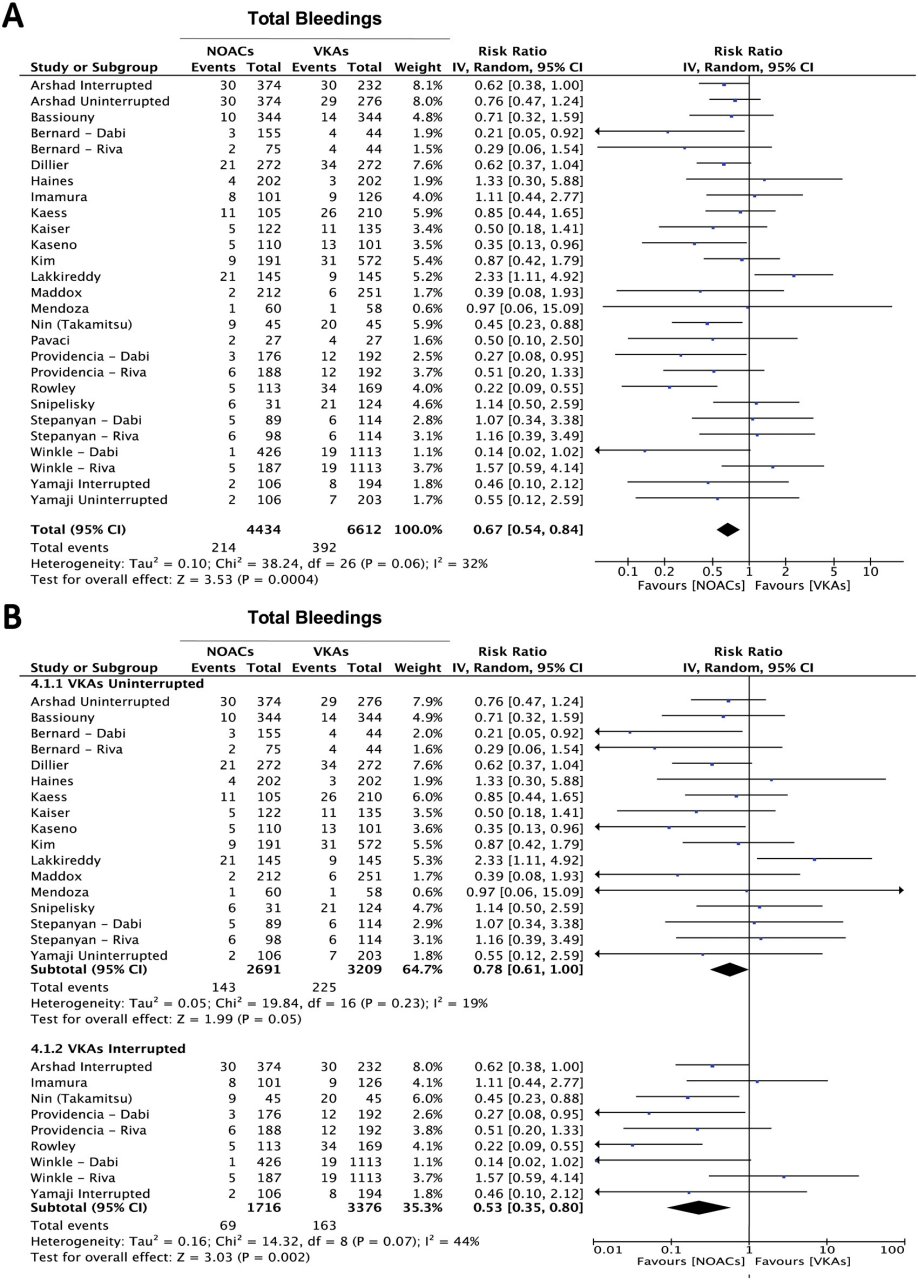
Meta-analysis of difference in total bleeding events. Panel A: forest plot and summary effect of the difference in the incidence of total bleedings. Panel B: difference in the total bleeding events is shown in the subgroups of patients undergoing interrupted vs. uninterrupted periprocedural VKAs.

Analyzing the results from all studies reporting this endpoint, a lower incidence of total bleeding events was registered in the NOACs-treated arm (RR = 0.67, 95% CI 0.54–0.84; p = 0.0004) (Fig 4A). A subgroup analysis showed a substantial difference in the incidence of total bleedings in favor of the NOACs-treated arm both in the subgroup of studies in which the VKAs treatment had been interrupted and a heparin bridging had been performed during the periprocedural phase (RR = 0.53 95% CI 0.35–0.80; p = 0.002) (Fig 4B) and in the subgroup with no periprocedural VKAs interruption, where a much narrower but still statistically significant difference was observed (RR = 0.78 95% CI 0.61–1.00; p = 0.05) (Fig 4B). Furthermore, since 22 studies had used dabigatran and 6 rivaroxaban, we performed a further subgroup analysis, which did not show a substantial difference between the study subgroups using different NOACs (S1 Fig). Accordingly, comparison between the study subgroups didn’t show a statistically significant difference (p = 0.66).

**Fig 5:**
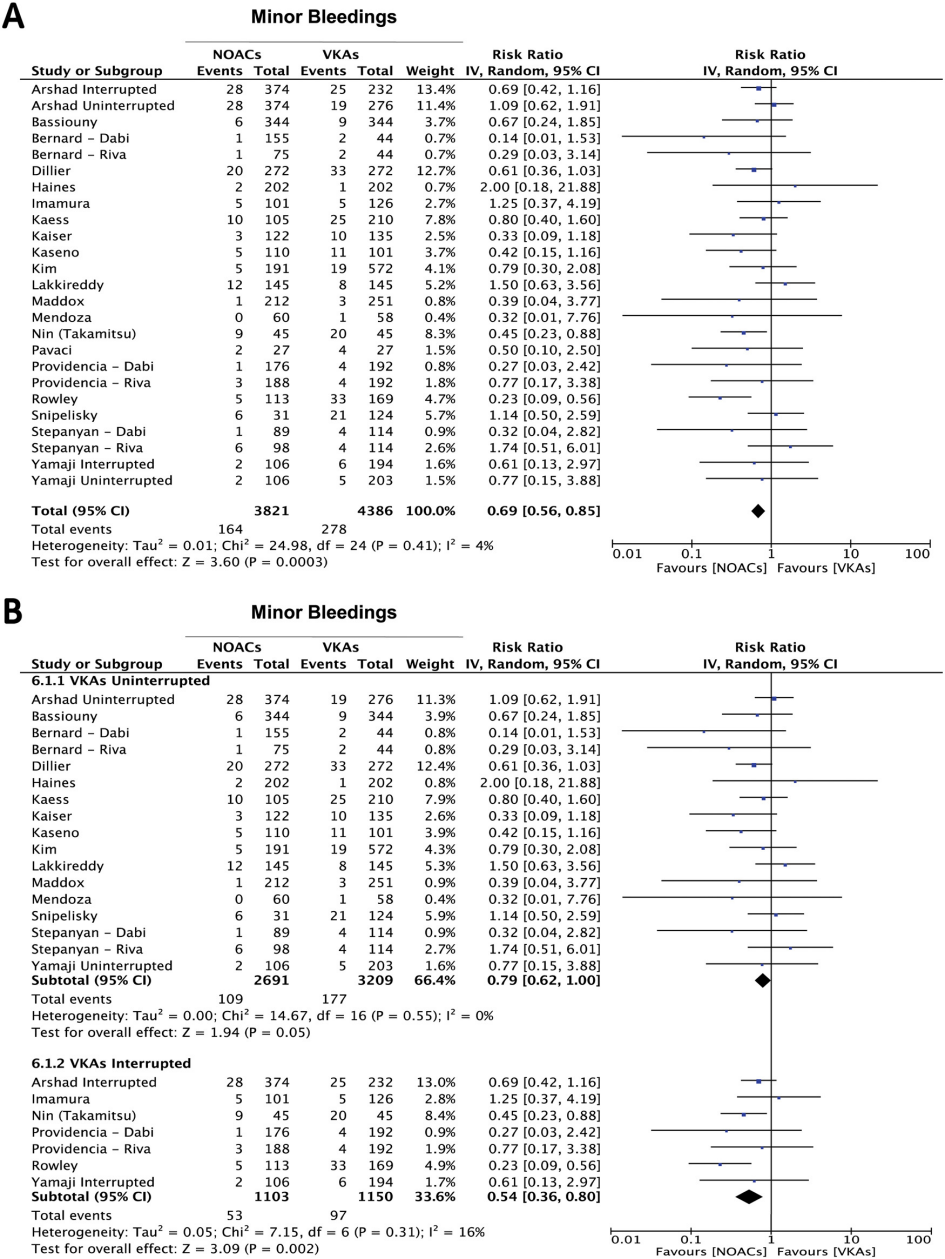
Meta-analysis of difference in minor bleeding events. Panel A: forest plot and summary effect of the difference in the incidence of minor bleedings. Panel B: difference in the minor bleeding events is shown in the subgroups of patients undergoing interrupted vs uninterrupted periprocedural VKAs.

**Fig 6:**
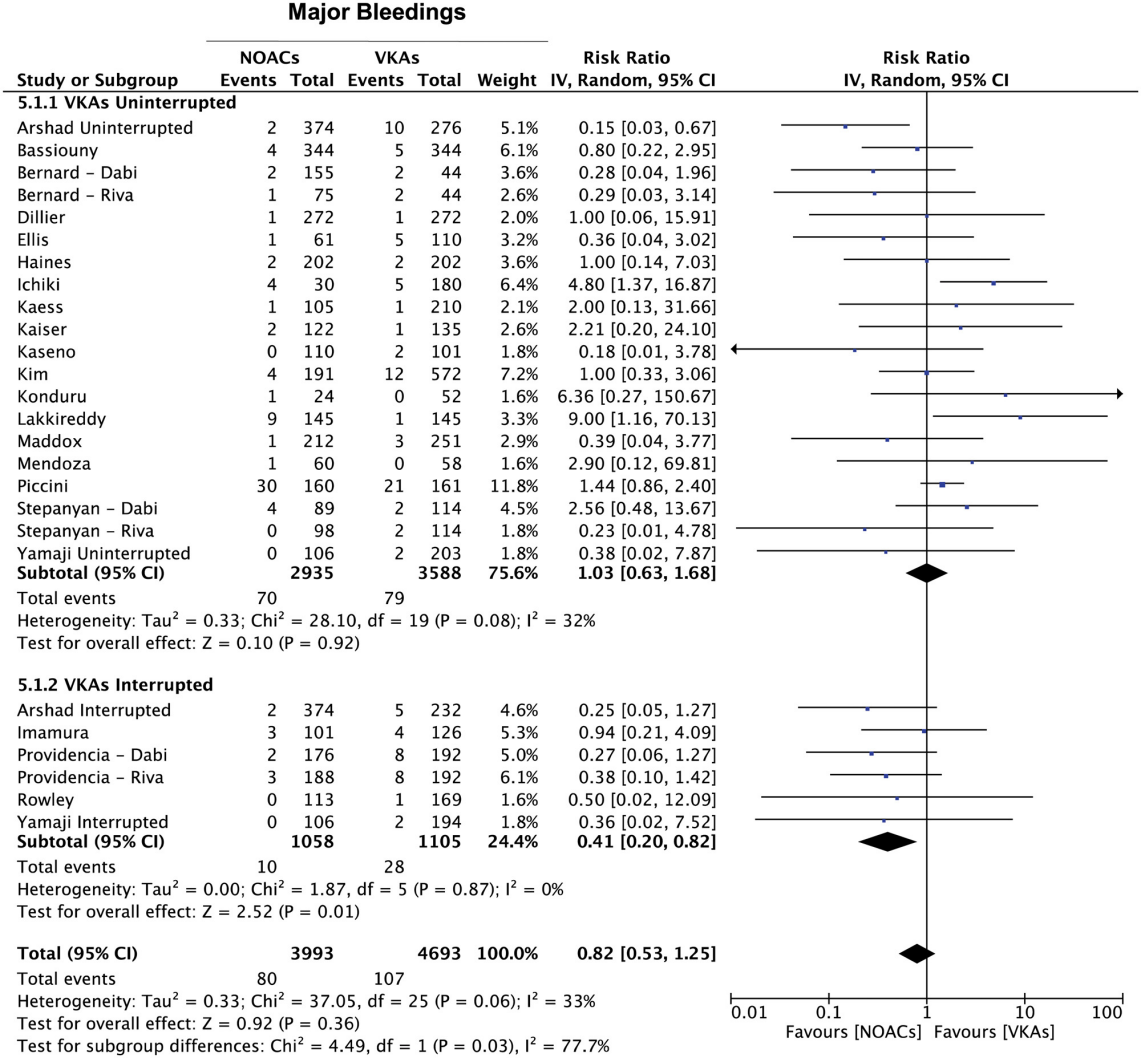
Meta-analysis of difference in major bleeding events. Forest plot and summary effect of the difference in the incidence of major bleedings within the subgroups of patients undergoing interrupted vs uninterrupted periprocedural VKAs. The bottom line reports the summary effect for both groups.

However, when the same comparison was performed after separating major (intracranial, gastrointestinal or any other type of bleedings requiring blood transfusion) from minor bleedings, we found a significantly lower incidence in minor bleedings in favor of the NOACs (RR = 0.69 95% CI 0.56–0.85; p = 0.0003) (Fig 5A), while no statistically significant difference in the incidence of major bleedings was evident, despite a clear trend in favor of the NOACs-treated arm (RR = 0.82 95% CI 0.53–1.25; p = 0.36) (Fig 6). Interestingly, interruption of VKAs during the periprocedural phase (with concomitant heparin bridging) was associated to a higher incidence of both minor (RR = 0.54 95% CI 0.36–0.80; p = 0.002)(Fig 5B) and major bleedings (RR = 0.41 95% CI 0.20–0.82; p = 0.01)(Fig 6) in comparison to the treatment with NOACs.

## Discussion

The present study is the largest and most comprehensive meta-analysis comparing the non-vitamin K antagonists oral anticoagulants to vitamin K antagonist oral anticoagulants in patients undergoing rhythm control management of atrial fibrillation by RFCA, with 25 selected studies that included 9881 cases. In contrast with a recently published meta-analysis, performed on a smaller group of studies [41], we found no significant difference in the incidence of total thromboembolic events between the NOACs-treated arm and the VKAs group. Foremost, the present meta-analysis is the first showing an advantage in favor of the VKAs-treated group on thromboembolic events in the subgroup of studies in which the VKAs treatment wasn’t interrupted in the periprocedural phase. Moreover, in the same study subgroup, interruption of VKAs with concomitant heparin bridging apparently exposed the patients to a higher bleeding risk. These latter results are not surprising, since a recent randomized international multicentre trial demonstrated a better outcome when the RFCA procedure was performed without VKAs discontinuation, over the alternative strategy of discontinuing VKAs with concomitant heparin bridging [42]. Previous meta-analyses performed on smaller number of studies, have shown a similar efficacy and safety profile of NOACs and VKAs [43,44,45]. In particular, Providencia et al. [44] performed a meta-analysis and reported no significant differences between dabigatran and warfarin for thromboembolic events (0.55% with dabigatran vs. 0.17% with warfarin, risk ratio 1.78). Similarly, Hohnloser et al. [45] reported no significant differences. With both these meta-analyses a numerically lower recurrence of thromboembolic events was observed within the warfarin-treated arm. Compared to previous meta-analyses, however, our study includes the largest number of studies and patients available so far assigned to a rhythm management strategy of atrial fibrillation, by means of RFCA, including dabigatran and rivaroxaban, while all previous analyses were mostly focused on dabigatran-treated patients. The novel finding of an increased incidence of thromboembolic events in patients undergoing RFCA in the NOACs-treatment arm could be related to the different pharmacodynamic profile of these two classes of oral anticoagulants. In fact, while the anticoagulant effect of VKAs gradually fluctuates over longer periods, warranting a seemingly stable anticoagulant effect on the short term in patients on target, the half-life of NOACs ranges 12–17h. Hence, their anticoagulant effect mostly vanishes within 24–36 hours from the last assumption. Therefore, the differences in the timing of administration of NOACs before and after RFCA, could have affected their efficacy in the periprocedural setting. In fact, in almost all studies, the NOAC was interrupted at least 12h before the RFCA procedure and started again the day after, allowing a short—yet not clinically irrelevant—window of suboptimal anticoagulation. This hypothesis is further supported by our findings in the subgroup analysis—which we could perform given the large number of studies included—showing that the superiority of VKAs in the prevention of thromboembolic events was less evident in the subgroup of studies in which the VKAs treatment was interrupted during the periprocedural phase. Altogether these findings highlight the importance of an optimal periprocedural coagulation, since thromboembolic events are concentrated around the time of rhythm conversion. At the same time, the higher bleeding rate observed with VKAs in the study subgroup with VKAs interruption and heparin bridging suggests that maintenance of a constant level of anticoagulation is of key importance in preventing dreadful bleeding events. Another possible explanation for the superiority observed with VKAs in the prevention of thromboembolic events is the chance to monitor their anticoagulant effect, while no validated method for assessing the anticoagulant efficacy of an ongoing treatment with NOACs is available yet. This fact could also potentially explain the higher bleeding rate when VKAs treatment was periprocedurally substituted by LMWH bridging, which cannot be monitored. Finally, another aspect to take into account is represented by the possible drug interactions of dabigatran, the NOAC used in many studies, with amiodarone and dronedarone. Unfortunately, the frequency of use of these both drugs in the selected studies is not known. However, since they are frequently used in patients undergoing a rhythm management strategy, we cannot exclude that they were largely used in the selected studies. The results of the present meta-analysis underline the importance of an optimal balance between risks and benefits in medicine. In particular, during the peri-procedural phase a higher (bleeding-) risk propensity is justified by the substantial prevention of thromboembolic events. However, these findings shouldn’t be generalized to other clinical settings. In fact, during the post-ablation phase, a lower thromboembolic risk probably doesn’t justify the routinely use of oral anticoagulation. A recent analysis from a large Danish cohort apparently supports this concept. In fact, use of oral anticoagulation beyond the third month after RFCA was associated with a 1.8% increase of serious bleedings, with only 0.6% reduction of the thromboembolic risk [46].

## Limitations

Given the large number of non-randomized studies included in the present meta-analysis, we cannot exclude the risk of a selection bias. In fact only a minority of trials were randomized or prospective and a blinded analysis and central adjudication of endpoints was performed in a minority of the included studies. Nevertheless, the inclusion of non-randomized studies in a meta-analysis is recommended by many authors since it helps in generalizing the conclusions to the daily clinical routine. Furthermore, since the NOAC was interrupted 12–30h before the RFCA procedure and started again the day after in almost all studies, no comparison could be performed distinguishing interrupted and non-interrupted NOAC treatment. Finally, despite the studies were generally well balanced between the treatment arms, especially regarding the predicted thromboembolic risk and the bleeding risk, these last data were heterogeneously reported in the selected studies, making impossible to exploit them as a moderator variable for a meta-regression.

## Conclusions

Notwithstanding the above cited potential limitations, the present meta-analysis—the largest and most comprehensive available to date—shows no significant difference for the prevention of thromboembolic complications in patients with atrial fibrillation managed with RFCA, although a trend is evident in favor of the VKAs-treated group. Interestingly, a significantly lower incidence of thromboembolic complications was found in favor of the VKAs-treated arm in the subgroup of studies in which the VKAs treatment was not interrupted in the periprocedural phase, although this was paid back with a slightly higher incidence of bleeding complications. Besides confirming the safety profile of NOACs, our results also highlight the importance of the optimal periprocedural management of anticoagulation. In this respect, the design of a prospective trial to evaluate the optimal periprocedural anticoagulation is desirable, to test efficacy and safety of uninterrupted periprocedural VKA versus uninterrupted periprocedural NOAC treatment in patients with atrial fibrillation undergoing a RFCA procedure.

## Supporting Information

S1 Fig Dabigatran and Rivaroxaban Subgroups. Subgroup analysis comparing the study subgroup using dabigatran to the study subgroup using rivaroxaban.
(EPS)

S1 Table Prisma 2009 checklist. (DOC)

S2 Table Baseline risk and events’ list. CHADS_2_ or the CHA_2_DS_2_-VASc score and the HAS-BLED score, along with the number of events for each single study.
(DOC)

S3 Table Endpoints’ definition. Definition of thromboembolic and bleeding complications.
(DOC)

## References

[1] Stewart S, Hart CL, Hole DJ, McMurray JJ. Population prevalence, incidence, and predictors of atrial fibrillation in the Renfrew/Paisley study. Heart. 2001;86: 516–521. 11602543 10.1136/heart.86.5.516 PMC1729985

[2] Lloyd-Jones D, Adams R, Carnethon M, De Simone G, Ferguson TB, Flegal K, et al Heart disease and stroke statistics—2009 update: A report from the AHA Statistics Committee and Stroke Statistics Subcommittee. Circulation. 2009;119: 480–486. 10.1161/CIRCULATIONAHA.108.191259 19171871

[3] Hart RG, Pearce LA, Aguilar MI. Meta-analysis: antithrombotic therapy to prevent stroke in patients who have nonvalvular atrial fibrillation. Annals of Internal Medicine. 2007;146: 857–867. 17577005 10.7326/0003-4819-146-12-200706190-00007

[4] Samsa GP, Matchar DB, Goldstein LB, Bonito AJ, Lux LJ, Witter DM, et al Quality of anticoagulation management among patients with atrial fibrillation: results of a review of medical records from 2 communities. Arch Intern Med. 2000;160: 967–973. 10761962 10.1001/archinte.160.7.967

[5] Connolly SJ, Ezekowitz MD, Yusuf S, Eikelboom J, Oldgren J, Parekh A, et al Dabigatran versus warfarin in patients with atrial fibrillation. N Engl J Med. 2009;361: 1139–1151. 10.1056/NEJMoa0905561 19717844

[6] Patel MR, Mahaffey KW, Garg J, Pan G, Singer DE, Hacke W, et al Rivaroxaban versus warfarin in nonvalvular atrial fibrillation. N Engl J Med. 2011;365: 883–891. 10.1056/NEJMoa1009638 21830957

[7] Granger CB, Alexander JH, McMurray JJ, Lopes RD, Hylek EM, Hanna M, et al Apixaban versus warfarin in patients with atrial fibrillation. N Engl J Med. 2011;365: 981–992. 10.1056/NEJMoa1107039 21870978

[8] Camm JA, Lip GYH, De Caterina R, Savelieva I, Atar D, Hohnloser SH, et al 2012 focused update of the ESC Guidelines for the management of atrial fibrillation: an update of the 2010 ESC Guidelines for the management of atrial fibrillation. Eur Heart J. 2012;33: 2719–2747. 10.1093/eurheartj/ehs253 22922413

[9] De Rosa S, Caiazzo G, Torella D, Indolfi C. Intracoronary abciximab reduces death and major adverse cardiovascular events in acute coronary syndromes: A meta-analysis of clinical trials. Int J Cardiol. 2012;168: 1298–1305. 10.1016/j.ijcard.2012.12.003 23273690

[10] Moher D, Liberati A, Tetzlaff J, Altman DG. The PRISMA Group. Preferred reporting items for systematic reviews and meta-analysis: the PRISMA statement. PLoS Med. 2009;6:e1000097 10.1371/journal.pmed.1000097 19621072 PMC2707599

[11] DerSimonian R, Laird N. Meta-analysis in clinical trials. Control Clin Trials. 1986;7: 177–188. 3802833 10.1016/0197-2456(86)90046-2

[12] Mantel N, Haenszel W. Statistical aspects of the analysis of data from retrospective studies of disease. J Natl Cancer Inst. 1959;22: 719–748. 13655060

[13] De Rosa S, Caiazzo G, Torella D, Indolfi C. Letter to the editor: Intracoronary versus intravenous abciximab bolus administration. J Am Coll Cardiol. 2014;63: 1340–1341. 10.1016/j.jacc.2013.08.1662 24412448

[14] De Rosa S, Torella D, Caiazzo G, Giampà S, Indolfi C. Left radial access for percutaneous coronary procedures: from neglected to performer? A meta-analysis of 14 studies including 7,603 procedures. Int J Cardiol. 2014;171: 66–72. 10.1016/j.ijcard.2013.11.046 24331866

[15] Egger M, Davey Smith G, Schneider M, Minder C. Bias in meta-analysis detected by a simple graphical test. BMJ. 1997;315: 629–634. 9310563 10.1136/bmj.315.7109.629 PMC2127453

[16] Maddox W, Kay GN, Yamada T, Osorio J, Doppalapudi H, Plumb VJ, et al Dabigatran versus warfarin therapy for uninterrupted oral anticoagulation during atrial fibrillation ablation. J Cardiovasc Electrophysiol. 2013;24: 861–865. 10.1111/jce.12143 23577951

[17] Winkle RA, Mead RH, Engel G, Kong MH, Patrawala RA. Peri-procedural interrupted oral anticoagulation for atrial fibrillation ablation: comparison of aspirin, warfarin, dabigatran, and rivaroxaban. Europace. 2014;16: 1443–1449. 10.1093/europace/euu196 25115168 PMC4178475

[18] Ichiki H, Oketani N, Ishida S, Iriki Y, Okui H, Maenosono R, et al The incidence of asymptomatic cerebral microthromboembolism after atrial fibrillation ablation: comparison of warfarin and dabigatran. PACE. 2013;36: 1328–1335. 10.1111/pace.12195 23952291

[19] Piccini JP, Stevens SR, Lokhnygina Y, Patel MR, Halperin JL, Singer DE, et al Outcomes after cardioversion and atrial fibrillation ablation in patients treated with Rivaroxaban and Warfarin in the ROCKET AF Trial. J Am Coll Cardiol. 2013;61: 1998–2006. 10.1016/j.jacc.2013.02.025 23500298

[20] Kaseno K, Naito S, Nakamura K, Sakamoto T, Sasaki T, Tsukada N, et al Efficacy and safety of periprocedural dabigatran in patients undergoing catheter ablation of atrial fibrillation. Circ J. 2012;76: 2337–2342. 22785434 10.1253/circj.cj-12-0498

[21] Snipelisky D, Kauffman C, Prussak K, Johns G, Venkatachalam K, Kusumoto F. Comparison of bleeding complications post-ablation between warfarin and dabigatran. J Interv Card Electrophysiol. 2012;35: 29–33. 10.1007/s10840-012-9708-z 22869389

[22] Konduru SV, Cheema AA, Jones P, Li Y, Ramza B, Wimmer AP. Differences in intraprocedural ACTs with standardized heparin dosing during catheter ablation for atrial fibrillation in patients treated with dabigatran vs. patients on uninterrupted warfarin. J Interv Card Electrophysiol. 2012;35: 277–284. 10.1007/s10840-012-9719-9 23015216

[23] Kaiser DW, Streur MM, Nagarakanti R, Whalen SP, Ellis CR. Continuous warfarin versus periprocedural dabigatran to reduce stroke and systemic embolism in patients undergoing catheter ablation for atrial fibrillation or left atrial flutter. J Interv Card Electrophysiol. 2013;37: 241–247. 10.1007/s10840-013-9793-7 23625091

[24] Haines DE, Mead-Salley M, Salazar M, Marchlinski FE, Zado E, Calkins H, et al Dabigatran versus warfarin anticoagulation before and after catheter ablation for the treatment of atrial fibrillation. J Interv Card Electrophysiol. 2013;37: 233–239. 10.1007/s10840-013-9800-z 23740224

[25] Bernard M, Brabham W, Netzler P, Rowley C, Gold M, Leman R, et al Comparison of atrial fibrillation ablation bleeding and thrombotic complications with dabigatran, rivaroxaban and warfarin. J Am Coll Cardiol. 2013;61(Suppl):E276 24236310

[26] Kim JS, She F, Jongnarangsin K, Chugh A, Latchamsetty R, Ghanbari H, et al Dabigatran vs warfarin for radiofrequency catheter ablation of atrial fibrillation. Heart Rhythm. 2013;10: 483–489. 10.1016/j.hrthm.2012.12.011 23237911

[27] Yamaji H, Murakami T, Hina K, Higashiya S, Kawamura H, Murakami M, et al Usefulness of dabigatran etexilate as periprocedural anticoagulation therapy for atrial fibrillation ablation. Clin Drug Investig. 2013;33: 409–418. 10.1007/s40261-013-0081-1 23572324

[28] Mendoza I, Helguera M, Baez-Escudero J, Reina J, Pinski SL. Atrial fibrillation ablation on uninterrupted anticoagulation with dabigatran versus warfarin. Heart Rhythm. 2012;9(5Suppl 1):S270–1.

[29] Rowley CP, Bradford NS, Bernard ML, Sidney DS, Brabham WW, Netzler PC, et al Complications of atrial fibrillation ablation in patients anticoagulated with dabigatran compared to warfarin. Heart Rhythm. 2012;9 (5Suppl 1):S201.

[30] Pavaci H, Reents T, Ammar S, Fichtner S, Schoen P, Kathan S, et al Safety and efficacy of dabigatran in patients undergoing left atrial ablation procedures: a case-matched analysis. Eur Heart J. 2012;33: 60–61. 10.1093/eurheartj/ehr257 21804108

[31] Ellis CR, Streur MM, Nagarakanti R. Safety and efficacy of dabigatran versus warfarin in patients undergoing left atrial catheter ablation. Heart Rhythm. 2012;9: S421.

[32] Stepanyan G, Badhwar N, Lee RJ, Marcus GM, Lee BK, Tseng ZH, et al Safety of new oral anticoagulants for patients undergoing atrial fibrillation ablation. J Interv Card Electrophysiol. 2014;40: 33–38. 10.1007/s10840-014-9888-9 24643666

[33] Arshad A, Johnson CK, Mittal S, Buch E, Hamam I, Tran T, et al Comparative Safety of Periablation Anticoagulation Strategies for Atrial Fibrillation: Data from a Large Multicenter Study. Pacing Clin Electrophysiol. 2014;37: 665–673. 10.1111/pace.12401 24797604 PMC4253027

[34] Dillier R, Ammar S, Hessling G, Kaess B, Pavaci H, Buiatti A, et al Safety of continuous periprocedural rivaroxaban for patients undergoing left atrial catheter ablation procedures. Circ Arrhythm Electrophysiol. 2014;7: 576–582. 10.1161/CIRCEP.114.001586 24970293

[35] Kaess BM, Ammar S, Reents T, Dillier R, Lennerz C, Semmler V, et al Comparison of safety of left atrial catheter ablation procedures for atrial arrhythmias under continuous anticoagulation with apixaban versus phenprocoumon. Am J Cardiol. 2015;115: 47–51. 10.1016/j.amjcard.2014.10.005 25456870

[36] Nin T, Sairaku A, Yoshida Y, Kamiya H, Tatematsu Y, Nanasatur M, et al A randomized controlled trial of dabigatran versus warfarin for periablation anticoagulation in patients undergoing ablation of atrial fibrillation. PACE. 2013;36: 172–179. 10.1111/pace.12036 23121681

[37] Lakkireddy D, Reddy YM, Di Biase L, Vanga SR, Santangeli P, Swarup V, et al Feasibility and safety of dabigatran versus warfarin for periprocedural anticoagulation in patients undergoing radiofrequency ablation for atrial fibrillation. J Am Coll Cardiol. 2012;59: 1168–1174. 10.1016/j.jacc.2011.12.014 22305113

[38] Imamura K, Yoshida A, Takei A, Fukuzawa K, Kiuchi K, Takami K, et al Dabigatran in the peri-procedural period for radiofrequency ablation of atrial fibrillation: efficacy, safety, and impact on duration of hospital stay. J Interv Card Electrophysiol. 2013;37: 223–231. 10.1007/s10840-013-9801-y 23585240 PMC3738875

[39] Bassiouny M, Saliba W, Rickard J, Shao M, Sey A, Diab M, et al Use of dabigatran for periprocedural anticoagulation in patients undergoing catheter ablation for atrial fibrillation. Circ Arrhythm Electrophysiol. 2013;6: 460–466. 10.1161/CIRCEP.113.000320 23553523 PMC3688655

[40] Providencia R, Marijon E, Albenque J, Combes S, Combes N, Jourda F, et al Rivaroxaban and dabigatran in patients undergoing catheter ablation of atrial fibrillation. Europace. 2014;16: 1137–1144. 10.1093/europace/euu007 24550347

[41] Sardar P, Nairooz R, Chatterjee S, Wetterslev J, Ghosh J, Aronow WS. Meta-analysis of risk of stroke or transient ischemic attack with dabigatran for atrial fibrillation ablation. Am J Cardiol. 2014;113: 1173–1177. 10.1016/j.amjcard.2013.12.027 24513472

[42] Di Biase L, Burkhardt JD, Santangeli P, Mohanty P, Sanchez JE, Horton R, et al Periprocedural stroke and bleeding complications in patients undergoing catheter ablation of atrial fibrillation with different anticoagulation management: results from the role of Coumadin in preventing thromboembolism in atrial fibrillation (AF) patients undergoing catheter ablation (COMPARE) Randomized Trial. Circulation. 2014;129: 2638–2644. 10.1161/CIRCULATIONAHA.113.006426 24744272

[43] Shurrab M, Morillo CA, Schulman S, Kansal N, Danon A, Newman D, et al Safety and Efficacy of Dabigatran Compared With Warfarin for Patients Undergoing Radiofrequency Catheter Ablation of Atrial Fibrillation: A Meta-analysis. Can J Cardiol. 2013;29: 1203–1210. 10.1016/j.cjca.2013.07.005 23993352

[44] Providência R, Albenque JP, Combes S, Bouzeman A, Casteigt B, Combes N, et al Safety and efficacy of dabigatran versus warfarin in patients undergoing catheter ablation of atrial fibrillation: a systematic review and meta-analysis. Heart. 2014;100: 324–335. 10.1136/heartjnl-2013-304386 23878175 PMC3913219

[45] Hohnloser SH, Camm AJ. Safety and efficacy of dabigatran etexilate during catheter ablation of atrial fibrillation: a meta-analysis of the literature. Europace. 2013;15: 1407–1411. 10.1093/europace/eut241 23954917

[46] Karasoy D, Gislason GH, Hansen J, Johannessen A, Køber L, Hvidtfeldt M, et al Oral anticoagulation therapy after radiofrequency ablation of atrial fibrillation and the risk of thromboembolism and serious bleeding: long-term follow-up in nationwide cohort of Denmark. Eur Heart J. 2015;36: 307–315. 10.1093/eurheartj/ehu421 25368205

