# Supplementary material for: Efficacy and Safety of Non-Vitamin K Antagonist Oral Anticoagulants versus Vitamin K Antagonist Oral Anticoagulants in Patients Undergoing Radiofrequency Catheter Ablation of Atrial Fibrillation: A Meta-Analysis
Source: PLoS One. 2015 May 14;10(5):e0126512. doi: 10.1371/journal.pone.0126512 (PMC4431735; doi:10.1371/journal.pone.0126512)
Supplement: S2 Table — CHADS2 or the CHA2DS2-VASc score and the HAS-BLED score, along with the number of events for each single study. (DOC) [file pone.0126512.s003.doc]

**Table 2. Baseline risk and events’ list.**

| **STUDY** | **STUDY SUBGROUP** | **N** | **THROMBO-EMBOLIC RISK SCORE** | **HAEMORRAGIC RISK SCORE (HAS-BLED)** | **TOTAL ISCHAEMIC EVENTS** | **STROKE** | **TIA/SCI** | **SE/PE** | **Major bleeding** | **Minor Bleeding** | **Total Bleeding** |
| --- | --- | --- | --- | --- | --- | --- | --- | --- | --- | --- | --- |
| Kaseno | NOAC | 110 | CHADS_2_score  0 - 67(61%)  1 - 34(31%) | 0.5±0.7 | 1 | 0 | 1 | 0 | 0 | 5 | 5 |
|  | VKA | 101 | CHADS_2_score  0 -46(45%)  1 - 41(41%) | 0.6±0.6 | 1 | 0 | 1 | 0 | 2 | 11 | 13 |
| Lakkireddy | NOAC | 145 | CHADS_2_score  0 - 50 (35%)  1- 62 (43%)  ≥ 2 - 33 (23%)  CHA_2_DS_2_-VASc score 1.6±1.4 | 1.2±0.9 | 3 | 0 | 3 | 0 | 9 | 12 | 21 |
|  | VKA | 145 | CHADS_2_score  0 - 58 (40%)  1 - 60 (41%)  ≥2 - 27 (19%)  CHA_2_DS_2_-VASc score  1.5±1.3 | 1.1±0.9 | 0 | 0 | 0 | 0 | 1 | 8 | 9 |
| Snipelisky | NOAC | 31 | CHADS_2_ score  0.84 | - | 0 | 0 | 0 | 0 | 0 | 6 | 6 |
|  | VKA | 125 | CHADS_2_ score  1.22 | - | 0 | 0 | 0 | 0 | 0 | 21 | 21 |
| Konduru | NOAC | 24 | - | - | 0 | 0 | 0 | 0 | 1 | - | - |
|  | VKA | 52 | - | - | 0 | 0 | 0 | 0 | 0 | - | - |
| Piccini | NOAC | 160 | CHADS_2_ score (median)  3 | - | 3 | - | - | - | 30 | - | - |
|  | VKA | 161 | CHADS_2_ score (median)  3 | - | 3 | - | - | - | 21 | - | - |
| Ichiki | NOAC | 30 | CHADS_2_ score  1.1±1.1 | - | 8 | 0 | 8 | 0 | 4 | - | - |
|  | VKA | 180 | CHADS_2_ score  1±1 | - | 18 | 0 | 18 | 0 | 5 | - | - |
| Kaiser | NOAC | 122 | CHADS_2_ score (mean)  1.2±1  CHADS_2_ score  ≤1 78 (64%) CHA_2_DS_2_-VASc score  1.8±1.4 | - | 3 | 1 | 1 | 1 | 2 | 3 | 5 |
|  | VKA | 135 | CHADS_2_ score (mean)  1.6±1  CHADS_2_ score ≤1  58 (43%)  CHA_2_DS_2_-VASc score  2.5±1.4 | - | 1 | 0 | 0 | 1 | 1 | 10 | 11 |
| Haines | NOAC | 202 | CHA_2_DS_2_-VASc score  1.6+-1.3 | - | 2 | 1 | 1 | 0 | 2 | 2 | 4 |
|  | VKA | 202 | CHA_2_DS_2_-VASc score  1.9+-1.4 | - | 0 | 0 | 0 | 0 | 2 | 1 | 3 |
| Imamura | NOAC | 101 | CHADS_2_ score  0.9±0.9  CHA_2_DS_2_-VASc score  1.6±1.2 | - | 1 | 0 | 1 | 0 | 3 | 5 | 8 |
|  | VKA | 126 | CHADS_2_ score  1.1±1.0.  CHA_2_DS_2_-VASc score  1.8±1.2 | - | 0 | 0 | 0 | 0 | 4 | 5 | 9 |
| Maddox | NOAC | 212 | CHADS_2_ score  0.92±0.88  CHA_2_DS_2_-VASc score  1.73±1.45 | - | 1 | 0 | 1 | 0 | 1 | 1 | 2 |
|  | VKA | 251 | CHADS_2_ score  0.92±0.85  CHA_2_DS_2_-VASc score  1.69±1.33 | - | 0 | 0 | 0 | 0 | 3 | 3 | 6 |
| Nin | NOAC | 45 | CHADS_2_ score  (0–1) - 37 (82%)  2 - 5 (11%) | - | 0 | 0 | 0 | 0 | 0 | 9 | 9 |
|  | VKA | 45 | CHADS_2_ score  (0–1) - 36 (80%)  2 - 6 (13%) | - | 1 | 0 | 0 | 1 | 0 | 20 | 20 |
| Bernard  (Dabigatran) | NOAC | 155 | CHA_2_DS_2_-VASc score  (mean)  2 | - | 0 | 0 | 0 | 0 | 2 | 1 | 3 |
|  | VKA | 44 | CHA_2_DS_2_-VASc score  (mean)  2.68 | - | 0 | 0 | 0 | 0 | 2 | 2 | 4 |
| Bernard  (Rivaroxaban) | NOAC | 75 | CHA_2_DS_2_-VASc score  (mean)  2.01 | - | 0 | 0 | 0 | 0 | 1 | 1 | 2 |
|  | VKA | 44 | CHA_2_DS_2_-VASc score  (mean)  2.68 | - | 0 | 0 | 0 | 0 | 2 | 2 | 4 |
| Kim | NOAC | 191 | CHADS_2_ score  1.0± 0.9  CHA_2_DS_2_-VASc score  1.6±1.3 | 1.0 +-0.9 | 0 | 0 | 0 | 0 | 4 | 5 | 9 |
|  | VKA | 572 | CHADS_2_ score  1.1±1.0  CHA_2_DS_2_-VASc score  1.7±1.3 | 1.1 +-0.9 | 0 | 0 | 0 | 0 | 12 | 19 | 31 |
| Bassiouny | NOAC | 344 | CHADS_2_ score  0- 135(39.2%)  1- 134(39%)  ≥ 2 - 75(21.8%) | - | 1 | 0 | 0 | 1 | 4 | 6 | 10 |
|  | VKA | 344 | CHADS_2_ score  0 - 137(39.8%)  1 - 137 (39.8%)  ≥2 - 70(20.3%) | - | 0 | 0 | 0 | 0 | 5 | 9 | 14 |
| Yamaji - interrupted VKA | NOAC | 106 | CHADS_2_ score  0- 38 (36%)  1 - 42 (40%)  ≥2 - 26 (24%)  CHA_2_DS_2_-VASc score  1.8 ± 1.6 | - | 0 | 0 | 0 | 0 | 0 | 2 | 2 |
|  | VKA | 194 | CHADS_2_ score  0- 66 (34%)  1 - 83 (43%)  ≥2 - 45 (23%)  CHA_2_DS_2_-VASc score  1.7 ± 1.4 | - | 0 | 0 | 0 | 0 | 2 | 6 | 8 |
| Yamaji - uninterrupted VKA | NOAC | 106 | CHADS_2_ score  0- 38 (36%)  1- 42 (40%)  ≥2- 26 (24%)  CHA_2_DS_2_-VASc score  1.8 ± 1.6 | - | 0 | 0 | 0 | 0 | 0 | 2 | 2 |
|  | VKA | 203 | CHADS_2_ score  0 - 65 (32%)  1 - 79 (39%)  ≥2 - 59 (29%)  CHA_2_DS_2_-VASc score  1.6 ± 1.5 | - | 0 | 0 | 0 | 0 | 2 | 5 | 7 |
| Mendoza | NOAC | 60 | CHADS_2_ score (mean)  1.32 | 1.47 | 0 | - | - | - | 1 | 0 | 1 |
|  | VKA | 58 | CHADS_2_ score (mean)  1.29 | 1.63 | 1 | - | - | - | 0 | 1 | 1 |
| Rowley | NOAC | 113 | CHADS_2_ score  1.3 ± 1 | - | 2 | 1 | 1 | 0 | 0 | 5 | 5 |
|  | VKA | 169 | CHADS_2_ score  1.3 ± 1 | - | 2 | 1 | 0 | 1 | 1 | 33 | 34 |
| Pavaci | NOAC | 27 | - | - | 1 | 1 | 0 | 0 | 0 | 2 | 2 |
|  | VKA | 27 | - | - | 0 | 0 | 0 | 0 | 0 | 4 | 4 |
| Ellis | NOAC | 61 | CHADS_2_ score  1.2 ± 0.2 | - | 2 | - | - | - | 1 | - | - |
|  | VKA | 110 | CHADS_2_ score  1.2 ± 0.2 | - | 1 | - | - | - | 5 | - | - |
| Stepanyan (Dabigatran) | NOAC | 89 | CHADS_2_ score  0 - 51.2%  1 - 44.3%  ≥2 - 5.5% | - | 1 | 0 | 1 | 0 | 4 | 1 | 5 |
|  | VKA | 114 | CHADS_2_ score  0 - 49.1%  1 - 44.1%  ≥2 - 6.8% | - | 1 | 0 | 1 | 0 | 2 | 4 | 6 |
| Stepanyan (Rivaroxaban) | NOAC | 98 | CHADS_2_ score  0 - 42.7%  1 - 48.8%  ≥2 - 8.5% | - | 0 | 0 | 0 | 0 | 0 | 6 | 6 |
|  | VKA | 114 | CHADS_2_ score  0 - 49.1%  1 - 44.1%  ≥2 - 6.8% | - | 1 | 0 | 1 | 0 | 2 | 4 | 6 |
| Providencia (Dabigatran) | NOAC | 176 | CHADS_2_ score  0.5±0.8  CHA_2_DS_2_-VASc score  1.2±1.2 | 0.7±0.8 | 1 | 0 | 1 | 0 | 2 | 1 | 3 |
|  | VKA | 192 | CHADS_2_ score  0.9±1  CHA_2_DS_2_-VASc score  1.8±1.4 | 1.1±0.9 | 4 | 4 | 0 | 0 | 8 | 4 | 12 |
| Providencia (Rivaroxaban) | NOAC | 188 | CHADS_2_ score  0.8±1  CHA_2_DS_2_-VASc score  1.5±1.3 | 1±0.9 | 2 | 1 | 1 | 0 | 3 | 3 | 6 |
|  | VKA | 192 | CHADS_2_ score  0.9±1  CHA_2_DS_2_-VASc score  1.8±1.4 | 1.1±0.9 | 4 | 4 | 0 | 0 | 8 | 4 | 12 |
| Arshad-  Interrupted VKA | NOAC | 374 | CHADS_2_ score  1.1 ± 1.1 | 2.2±1.3 | 1 | - | - | - | 2 | 28 | 30 |
|  | VKA | 232 | CHADS_2_ score  1.2 ± 1.1 | 2.8±1.0 | 2 | - | - | - | 5 | 25 | 30 |
| Arshad-  Uninterrupted VKA | NOAC | 374 | CHADS_2_ score  1.1 ± 1.1 | 2.2±1.3 | 1 | - | - | - | 2 | 28 | 30 |
|  | VKA | 276 | CHADS_2_ score  1.4 ± 1.4 | 2.1±1.5 | 4 | - | - | - | 10 | 19 | 29 |
| Winkle  (Dabigatran) | NOAC | 426 | CHADS_2_ score mean  1.28 ± 1.07  CHA_2_DS_2_-VASc score mean  2.13 ± 1.45 | - | 2 | 1 | 0 | 1 | - | - | 1 |
|  | VKA | 1113 | CHADS_2_ score mean  1.1 ± 1.05  CHA_2_DS_2_-VASc score mean  1.98.0 ± 1.44 | - | 5 | 4 | 1 | 0 | - | - | 19 |
| Winkle  (Rivaroxaban) | NOAC | 187 | CHADS_2_ score mean  1.55 ± 1.04  CHA_2_DS_2_-VASc score mean  2.48 ± 1.46 | - | 0 | 0 | 0 | 0 | - | - | 5 |
|  | VKA | 1113 | CHADS_2_ score mean  1.1 ± 1.05  CHA_2_DS_2_-VASc score mean  1.98.0 ± 1.44 | - | 5 | 4 | 1 | 0 | - | - | 19 |
| Dillier | NOAC | 272 | CHADS_2_ score mean  0.9 ± 0.8  CHA_2_DS_2_-VASc score mean  1.8 ± 1.4 | - | 0 | 0 | 0 | 0 | 1 | 20 | 21 |
|  | VKA | 272 | CHADS_2_ score mean  1 ± 0.9  CHA_2_DS_2_-VASc score mean  2 ± 1.5 | - | 0 | 0 | 0 | 0 | 1 | 33 | 34 |
| Kaess | NOAC | 105 | CHADS_2_ score mean  1.2 ± 1.1  CHA_2_DS_2_-VASc score mean  2.1 ± 1.8 | - | 0 | 0 | 0 | 0 | 1 | 10 | 11 |
|  | VKA | 210 | CHADS_2_ score mean  1 ± 0.9  CHA_2_DS_2_-VASc score mean  2 ± 1.5 | - | 0 | 0 | 0 | 0 | 1 | 25 | 26 |

**TIA/SCI= Transient Ischemic Attack/Silent Cerebral Ischemia ; SE/SP= Systemic Embolism/Pulmonary Embolism**
