# Supplementary material for: Efficacy and Safety of Non-Vitamin K Antagonist Oral Anticoagulants versus Vitamin K Antagonist Oral Anticoagulants in Patients Undergoing Radiofrequency Catheter Ablation of Atrial Fibrillation: A Meta-Analysis
Source: PLoS One. 2015 May 14;10(5):e0126512. doi: 10.1371/journal.pone.0126512 (PMC4431735; doi:10.1371/journal.pone.0126512)
Supplement: S3 Table — Definition of thromboembolic and bleeding complications. (DOC) [file pone.0126512.s004.doc]

**Table 3. Endpoints’ Definition.**

| **Study** | **Major bleeding** | **Minor bleeding** | **Thromboembolism** |
| --- | --- | --- | --- |
| Arshad | bleeding of any kind that necessitated blood transfusion or resulted in a 20% or greater fall in hematocrit | bleeding from any source requiring medical attention but not requiring transfusion or surgery . | stroke, or transient ischemic attack |
| Bassiouny | cardiac tamponade or hemopericardium  that required intervention or caused symptoms, excessive  bleeding (≥2 g/L decrease in hemoglobin or need for transfusion), hematoma  requiring intervention or additional hospitalization, significant  hemoptysis, hemothorax, or retroperitoneal bleeding. | minor hemorrhage  was defined as the occurrence of a hematoma or any bleeding that did not  require intervention or prolong hospitalization. | occurrence of ischemic stroke, transient ischemic attack, peripheral embolic events, or deep venous thrombosis |
| Bernard | pericardial tamponade, vascular hematoma or other bleeding reqiring transfusion | moderate to large pericardial effusion without tamponade, minor hematoma or other bleeding not requiring transfusion | - |
| Ellis | - | - | - |
| Haines** | pericardial tamponade or significant pericardial infusion requiring pericardiocentesis and any bleeding from the sites of catheter insertion or any other source resulting in transfusion of blood products or prolongation of hospitalization. | groin hematoma not requiring intervention | stroke, transient ischemic attack, and any other systemic embolus |
| Ichiki | cerebral microthromboembolism after AF ablation detected by MRI | - | - |
| Imamura | occurrence of cardiac tamponade, intracranial bleeding, fatal bleeding and any bleeding that resulted in a significant decrease in hemoglobin (≥2 g/dl) or required transfusion. | occurrence of hematoma or any bleeding that did not require surgery or transfusion. | occurrence of ischemic stroke, transient ischemic attack, or peripheral embolic events. |
| Kaiser | Bleeding with reduction in the packed cell volume (PCV) level of >8 %, or requiring transfusion of at least 2 units of blood, or symptomatic bleeding in a critical area or organ). | hematoma at vascular access site without criteria for major bleeding. | stroke, transient ischemic attack, and any other systemic embolus |
| Kaseno | intracranial hemorrhage, fall in the hemoglobin level >2g/dl, or need for blood transfusion. | groin hematoma | stroke, transient ischemic attack, and any other systemic embolus |
| Kim | pericardial tamponade that required percutaneous or surgical drainage or repair and vascular complications such as arteriovenous fistula, pseudoaneurysm, or hematoma that required percutaneous/ surgical intervention. | small groin hematoma, any bleeding that did not require an intervention, or pericardial effusion without tamponade. | stroke, transient ischemic attack, and any other systemic embolus |
| Konduru** | pericardial tamponade, bleeding requiring transfusion or extending the hospital stay beyond 24 h. | - | stroke, transient ischemic attack |
| Lakkireddy* | any bleeding requiring blood transfusion, hematomas requiring surgical intervention, and pericardial effusions requiring drainage. | small hematomas and pericardial effusions not requiring an intervention. | cerebrovascular accidents and transient ischemic attacks |
| Maddox* | - | - | - |
| Mendoza* | - | - | - |
| Nin | - | Bleeding from any source, observed within 48 hours after the ablation procedure, requiring neither a transfusion nor surgery. | - |
| Pavaci | - | - | - |
| Piccini** | As previously described^6^ | As previously described^6^ | As previously described^6^ |
| Providencia | cardiac tamponade, bleeding requiring intervention or transfusion, massive haemoptysis, haemothorax, retroperitoneal bleeding, or any other life-threatening bleed leading to prolongation of hospitalization | puncture site bleeding, thigh ecchymosis or hematoma, pericardial effusion with no haemodynamic compromise, minor gastrointestinal bleeding, epistaxis, or any bleeding treated conservatively with no need for transfusion, surgery, or prolonged hospitalization | stroke, transient ischemic attack, systemic or pulmonary embolism |
| Rowley | life threatening bleeding | non- life threatening bleeding | stroke, transient ischemic attack or systemic embolism |
| Snipelisky* | hemorrhage requiring blood products or the need for vascular intervention | prolonged bleeding from the catheter insertion site requiring reapplication of femoral artery compression assistance device. | - |
| Stepanyan | a significantly pericardial effusion detected by transthoracic echocardiogram (TTE), cardiac tamponade requiring drainage, any bleeding requiring transfusion or prolonging hospital stay | groin hematoma or any identified pseudoaneurysm not requiring surgery | stroke, transient ischemic attack, systemic or pulmonary embolism |
| Winkle | - | - | stroke, transient ischemic attack, systemic or pulmonary embolism |
| Yamaji | bleeding requiring blood transfusion, haematomas requiring surgical intervention, and cardiac tamponade requiring drainage. | small hematomas and pericardial effusions not requiring drainage. | stroke, transient ischemic attack, pulmonary embolism and deep venous embolism |
| Dillier* | bleeding requiring blood transfusion, retroperitoneal bleedings, hematomas requiring surgical intervention, pericardial effusions requiring drainage or surgical intervention and intracranial hemorrhage. | hematomas (>5 cm) not requiring blood transfusion or surgical intervention and pericardial effusions (>5 mm) not requiring an intervention. | cerebrovascular accidents and transient ischemic attacks. Deep vein thrombosis and pulmonary embolism. Peripheral arterial embolism. |
| Kaess* | bleeding requiring blood transfusion, retroperitoneal bleedings, hematomas requiring surgical intervention, pericardial effusions requiring drainage or surgical intervention and intracranial hemorrhage. | hematomas (>5 cm) not requiring blood transfusion or surgical intervention and pericardial effusions (>5 mm) not requiring an intervention. | cerebrovascular accidents and transient ischemic attacks. Deep vein thrombosis and pulmonary embolism. Peripheral arterial embolism. |

* Uninterrupted NOAC

** < 50 % of patients on continuous NOAC
